# The Educational Situation Quality Model: Recent Advances

**DOI:** 10.3389/fpsyg.2018.00328

**Published:** 2018-03-14

**Authors:** Fernando Doménech-Betoret

**Affiliations:** Developmental and Educational Psychology, Jaume I University, Castellón de la Plana, Spain

**Keywords:** educational model, research in the classroom, teaching–learning process, formative evaluation, learning outcomes

## Abstract

The purpose of this work was to present an educational model developed in recent years entitled the “The Educational Situation Quality Model” (MOCSE, acronym in Spanish). MOCSE can be defined as an instructional model that simultaneously considers the teaching-learning process, where motivation plays a central role. It explains the functioning of an educational setting by organizing and relating the most important variables which, according to the literature, contribute to student learning. Besides being a conceptual framework, this model also provides a methodological procedure to guide research and to promote reflection in the classroom. It allows teachers to implement effective research-action programs to improve teacher–students satisfaction and learning outcomes in the classroom context. This work explains the model’s characteristics and functioning, recent advances, and how teachers can use it in an educational setting with a specific subject. This proposal integrates approaches from several relevant psycho-educational theories and introduces a new perspective into the existing literature that will allow researchers to make progress in studying educational setting functioning. The initial MOCSE configuration has been refined over time in accordance with the empirical results obtained from previous research, carried out within the MOCSE framework and with the subsequent reflections that derived from these results. Finally, the contribution of the model to improve learning outcomes and satisfaction, and its applicability in the classroom, are also discussed.

## Introduction

Most of the educational models published in the educational literature to date have at least one of these three major deficiencies: (a) offer a reductionist view; i.e., models with this limitation are unable to capture the whole teaching-learning process, and provide only a partial and reductionist view of how an educational setting operates; (b) have a limited applicability, because they do not provide a useful methodology to apply their conceptual framework in the classroom; (c) are outdated, because most existing models do not take into account important psycho-educational findings reported in the literature of the 21st century. The “Educational Situation Quality Model” (MOCSE, acronym in Spanish), created by Doménech (2006, 2007, 2011a,b, 2012, 2013) in the last decade, overcomes the three above-mentioned limitations. Apart from being a conceptual framework, it provides a methodological procedure to guide research and to promote reflection in the classroom, which allows teachers to implement effective research-action programs to improve learning outcomes and teacher-students satisfaction. Unlike other educational models, MOCSE uses motivation (intentionality) as a central element, and offers an integrative and updated perspective of the teaching–learning process undertaken by the teacher and students in an educational setting.

**FIGURE 1:**
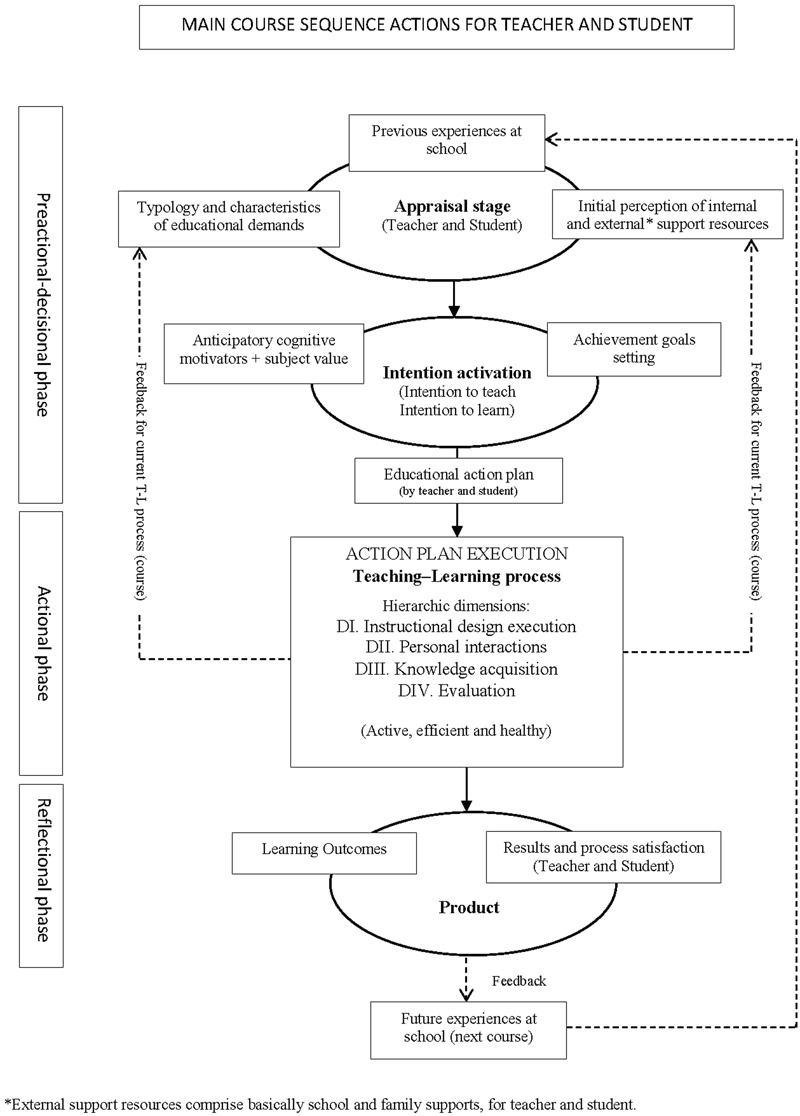
Sequential representation of the Educational Situation Quality Model at the macrosystemic level (focusing on the subject/course).

MOCSE was designed by taking the structure, dimensions and variables identified and selected in the Educational Situation Instructional Model as a starting point (Rivas, 1997, 2003). We found it valuable how this model organizes, from an integrative view, the most important variables of the teaching-learning process (see the MOCSE actional phase in the current paper for more details). Basically, in our proposal, the Educational Situation Instructional Model (Rivas, 1997, 2003) was reviewed and dimensions reorganized. That is, Dimension I (Intentionality) and Dimension II (Instructional design) are included as components of the MOCSE pre-actional decisional phase, and the other dimensions, plus instructional design execution, make up the actional phase (more explanations are provided below when our proposal is described). Contributions from other models and relevant scientific theories on teaching-learning processes were also taken into account. The initial MOCSE configuration has been refined over time in accordance with the empirical results obtained from the research carried out within the MOCSE framework and with the subsequent reflections that derived from these results. The updated proposal for a macrosystem approach (subject/course) is presented in **Figure 1**.

### The Model’s Scientific Foundation

The presented model (MOCSE) began to take shape with the completion of my doctoral dissertation (Doménech, 1995) based on the Educational Situation Instructional Model (designed by Rivas, 1997, 2003; the MISE acronym in Spanish). The main objective of that and other parallel studies conducted (Martínez, 1995; Gómez, 1995; Descals, 1996) focused on the same model, and consisted in examining the factorial validity and reliability of MISE, as well as its predictive capacity over academic achievement in different subjects and at several levels of education. The obtained results, published in several scientific journals, revealed that: (a) the MISE model structure is made up of five broad dimensions (with its corresponding subdimensions) that systemically, sequentially and hierarchically organize the variables (referring to teacher, subject content and students) involved in the teaching-learning process, beginning with the formulation of objectives and concluding with the evaluation of students’ learning outcomes: DI, Intentionality (goals and motivation); DII, Instruction Design; DIII, Personal interactions; DIV, Knowledge Acquisition; DV, Evaluation (Doménech, 1991, 1995; Martínez, 1995; Descals, 1996); (b) the MISE dimensions, especially the variables from DIV (Knowledge Acquisition), were able to explain and predict academic achievement with both university (Doménech et al., 2004), and secondary students (Rivas et al., 1997); (c) data also revealed that students’ initial perception of the teaching–learning process, referring to the DI (Intentionality) and DII (instructional design or subject-course planning) MISE dimensions, had a positive and significant effect on academic achievement (Rivas et al., 1997); finally, (d) it is a useful tool to conduct a formative evaluation of the whole teaching–learning process since it allows perceptions from students and teacher perspective to be captured and compared across the same referents; i.e., the MISE dimensions (see Doménech and Descals, 2003). In conclusion, first MOCSE was designed by taking the Educational Situation Instructional Model as a starting point (Rivas, 1997, 2003) and, second, based on the above reasoning and findings, we can assert that our proposal (MOCSE), presented in the current article, is supported by a solid scientific foundation.

### Empirical Data to Support the Current Proposal

Besides the above-explained MOCSE’s scientific foundation, later research has provided the necessary feedback and support to continue refining and constructing the model (Doménech, 2006). Below we summarize the most important findings obtained in recent research that supports the hypothesized connections defended in the model.

First, we examined and simultaneously tested the relationships among personal variables (internal support resources), the expectations-subject value and students’ avoidance learning strategies with the SEM (Structural Equation Modeling) approach (see Doménech et al., 2014). The results revealed strong associations among students’ personal variables (internal supports), their motivation at the beginning of the academic year and the avoidance strategies they used during the learning process followed in the Educational Psychology subject matter. The fit indices values obtained for the optimized model, using the ML Robust method of estimation (Satorra-Bentler scaled *χ^2^* = 185.228; *p* = 0.0155, d.f. = 146; χ^2^/d.f. = 1.268; NNFI = 0.89; CFI = 0.91; IFI = 0.91; RMSEA = 0.044), indicated that the model fitted the data.

Second, based on the MOCSE model’s assumptions, in a recent study (Abellán, 2016) more connections among variables were examined and simultaneously tested using the SEM (Structural Equation Modeling) procedure. For example, the relationship among students’ initial perceptions of the curriculum (Block 1), their personal resources (Block 2), their initial expectations-value subject (Block 3) and avoidance strategies (Block 4) were examined. The sample comprises 797 Spanish secondary education students. The scales that referred to personal variables and expectancy-value beliefs were administered at the beginning of the course (after some days of class), and students’ learning strategies were measured at the end of the course. The results proved a number of associations and effects hypothesized in MOCSE among students’ personal (general academic self-efficacy and prior knowledge), curricular variables (methodology and evaluation), their expectations at the beginning of the academic year (achievement expectations, process expectations, subject value, expected effort), and the avoidance learning strategies (help seeking, participation during the classroom, effort and challenges, team work collaboration, following teacher directions, and doing the required tasks), used by students during the teaching-learning process. The fit indices values obtained for the optimized model by the ML estimation method (*χ^2^* = 686.934; *p* = 0.000; d.f. = 164; χ^2^/d.f. = 4.18; NFI = 0.892; NNFI = 0.902; CFI = 0.915; GFI = 0.906; RMSEA = 0.069) and the ML Robust estimation method (the scale of Satorra-Bentler, *χ^2^* = 644.036; *p* = 0.000; d.f. = 164; *χ^2^*/d.f. = 3.92; NFI = 0.890; NNFI = 0.902; CFI = 0.915; IFI = 0.916; RMSEA = 0.066), indicated that the model satisfactorily fitted the data. The psychometric properties of the used scales were calculated and other structural submodels were also tested in this study (Abellán, 2016). The scales for secondary students are found in Doménech and Abellán (2017).

Another study based on the Educational Situation Quality Model (MOCSE) was conducted by Lozano-Nomdedeu (2016) with secondary school students. It examined the relationship among students’ intention to learn (expectancies and subject value), avoidance strategies (help seeking, participation during the classroom, effort and challenges, team work collaboration, following teacher directions, and doing the required tasks) and academic achievement. A questionnaire was administered at the beginning of the course, after some days of class, to measure students’ intention to learn. Avoidance learning strategies were measured at the end of the course by the teacher. Academic achievement (final marks) was also reported at the end of the course. The results revealed significant and negative associations (a) between students’ initial motivation or intention to learn (result expectancy, subject value and process expectancy) and avoidance strategies (novelty, team collaboration, and class participation respectively), and (b) between avoidance strategies (help seeking, challenges, class participation, novelty, and team work collaboration, following teacher directions) and academic achievement.

## Model Description

MOCSE (Doménech, 2006, 2007, 2011a,b, 2012, 2013) is an instructional model that attempts to coherently explain the functioning of a formal educational situation by selecting and organizing the main variables involved in school learning, and the relationships between them.

From a systemic perspective, MOCSE contemplates different levels of analyses that can be examined and arranged from a short-term to a long-term process: Task, Didactic Unit, Subject/Course (course with a specific subject matter), etc. Thus we can examine the teaching–learning (hereinafter T–L) process from either a microsystem approach as we focus on a short instructional segment or short-term process (e.g., didactic unit), or a macrosystem approach as we focus on a subject matter or a long-term process (e.g., subject/course). Based on this systemic view, and by taking a didactic unit as the unity of analysis, we consider that a subject/course is composed of a number of didactic units, where each one is made up by three specific sequential phases responsible for the results obtained in a didactic unit: Pre-actional decisional phase, Actional phase (decisions execution), Reflectional phase. For didactic and simplification reasons, the T–L process, undertaken with a subject during a course, has also been divided into the same, but broader and more general phases that are responsible for the final results obtained in a subject. **Figure 1** illustrates the MOCSE configuration from a macrosystem perspective; i.e., in an attempt to explain how an educational situation operates with a specific subject during a course. As **Figure 1** displays, MOCSE is made up of four components of variables (appraisal stage, Intention activation, teaching–learning process and product) distributed into three sequential phases: Pre-actional decisional phase, Actional phase (decisions execution), Reflectional phase (see **Figure 1** for more details). This sequential organization was based on the Action-Control Theory (Heckhausen and Kuhl, 1985) and the Process Model of Student Motivation (Dörnyei, 2000).

The Action-Control Theory (Heckhausen and Kuhl, 1985) distinguishes between the pre-decisional and post-decisional phases of motivation. The pre-decisional phase (also known as “choice motivation”) is associated with the intention-formation process and is considered the decision-making stage of motivation. The postdecisional phase (also known as “executive motivation”) is the implementational stage of motivation and is related to the volitional aspects of goal pursuit. This phase involves the maintenance and control of the motivational state during the action implementation process. Based on the Action-Control Theory (Heckhausen and Kuhl’s, 1985), Dörnyei (2000) devised a model of student motivation called the Process Model of Student Motivation to illustrate the temporal conception of motivation. His model comprises three main phases (pre-actional, actional, and post-actional phases). The pre-actional phase is associated with the decision-making stage or “choice motivation,” the actional phase with volition or “executive motivation,” and the post-actional phase is related to the causal attributions formed about the extent to which the pursued goal has been reached.

Component 1“Appraisal stage” and component 2 “Intention activation” comprise the Pre-actional decisional phase. Component 3, “Teaching–Learning process” corresponds to the Actional phase. Finally, component 4 “Product” corresponds to the “Reflectional phase.” Components 1 and 2 (pre-actional decisional phase) deal with those psychological processes and variables that are involved in the teacher and students’ decision making, which are capable of predicting and explaining the actions performed by the subjects (the teacher and students) during the T–L process (actional phase). The decisions made in the pre-actional phase put the model into practice. During the pre-actional phase, the agents who intervene (the teacher and students) in the educational setting make periodical re-evaluations (reappraisals) about the requested demands, supports and resources that they will receive to meet these demands and to overcome the barriers and difficulties, etc., that they will face during the T–L process, which may lead to changes in the actions to be taken and, in turn, in the results. The decisions made at the start of the educational process will have more chances of fluctuating in a long instructional segment (e.g., a course) and fewer chances during a short one (e.g., a instructional unit). Finally, the agents involved (the teacher and students) evaluate and reflect on the obtained outcomes (reflectional phase) and they self-regulate to become more efficient in the next instructional segment (task, unit, thematic block, course, etc.).

In the points below, we justify and provide background to the presented MOCSE configuration and explain which theories feed it (see the summary in **Table 1**). Despite it being a model that simultaneously deals with the teacher and students, we center basically on students for simplification and space reasons as students have the element that has generated most of the research conducted to date within the MOCSE framework.

**Table 1:** A summary of the theories, models and principles that feed the Educational Situation Quality Model and the specific contributions considered from each one.

Main theories and models which have fed the Educational Situation Quality Model	Specific contributions
*Theory of Cognitive Appraisal* (Lazarus and Folkman, 1984).	- Primary and secondary appraisals and coping strategies used by individuals in stressful situations. Theory’s postulates applied at school context.
*The Job Demands-Resources Model (JD-R)* (Demerouti et al., 2001; Bakker and Demerouti, 2007).	- The role played by job demands and resources at work and their relationship with occupational wellbeing. Theory’s postulates applied at school context.
*Social Cognitive Theory* (Bandura, 1987, 1989).	- Self-reflection and self-regulation concepts. - Self-efficacy and expectations of success constructs. - The mediating psychological processes between self-efficacy beliefs and conduct.
*Achievement Goal Theory* (Dweck and Legget, 1988; Nicholls, 1989; Ames, 1992).	- Types of achievement goals adopted by students. - The connections between type of goal adopted by students to engage in a specific task/course and learning outcomes.
*Expectancy-Value Theory* (Wigfield and Eccles, 2000).	- Explanatory variables of the achievement motivation in education: Task value and expectancy of success.
*Educational Situation Instructional Model* (Rivas, 1997, 2003).	- Integrative treatment of key instructional elements: Teacher, content, students. - Quality indicators that intervene in the T–L process, under the teacher’s and students’ responsibility.
*The Control-value Theory of Achievement Emotions* (Pekrun, 2006; Pekrun et al., 2007)	- Explanatory framework for the activation and deactivation of the emotions that affect teachers and students.
*The Action-Control Theory* (Heckhausen and Kuhl, 1985)	- Temporal conception of motivation: pre-decisional (choice motivation) and post-decisional (executive motivation) phase of motivation.

## Pre-Actional Decisional Phase

### Component 1: Appraisal Stage

#### The Role of Students’ Previous Experiences in the T–L Process

Previous research has found that students’ previous school experiences will influence the way they face the current educational situation. Continued poor performance and negative performance feedback produce frustration and may consequently affect future outcomes (Weiner, 1986).

Based on Expectancy-value theory, Gorges and Kandler (2012) found that adults’ appraisal of a new learning opportunity is influenced by previous learning experiences at school, specifically by expectancy of success (self-concept), value (interest), and affective memories. They conclude that “school experience is an antecedent of adults learning because experiences and values stabilize over time” (p. 612). According to the attribution theory, students’ own perceptions or attributions regarding to why they succeeded or failed at the school determine the amount of effort they will engage in school task in the future.

In sum, students’ initial perception of the current T–L process may be conditioned by previous academic experiences or by the information that students already have about the teacher. It is expected that a background of success will contribute to a more positive perception than a background of failures. Similar reasoning is also plausible for teacher’s initial perception.

Based on these assumptions and findings, we defend that the variables which refer to student success and failure attributions, as well as affective memories about past academic experiences, should be taken into account. The same recommendation can be made for teachers.

#### Learning Context in Terms of Demands and Support Resources

Based on the Job Demands-Resources Model (JD-R) (Demerouti et al., 2001; Bakker and Demerouti, 2007), the learning context refers to the perception which the teacher and students have formed of the educational setting in terms of demands and supports/resources. Demands can become stressing if they require a great deal of effort for the subject to achieve it. Subsequent research (Lorente et al., 2008) has the need to consider both job and personal resources to amplify the model’s explicative capacity. Adapting and applying the JD-R model to the school context implies identifying what the specific demands and resources are for teachers and students in the school context. The learning environment quality depends to a great extent on the simultaneous presence of environmental challenge (basically through educational demands) and the environmental support provided (Shernoff et al., 2016). One way of create a challenging classroom environment is designing novel and attractive demands.

#### The Role of Educational Demands in the Teaching–Learning Process

According to the Job Demands-Resources Model (JD-R) (Demerouti et al., 2001; Bakker and Demerouti, 2007), job demands “are physical, psychological, social or organizational aspects of work that require physical and/or psychological effort (cognitive or emotional), and are associated with a certain physiological and/or psychological cost” (Bakker and Demerouti, 2007, p. 312). Demands can become stressing if they require a great deal of effort for the subject to meet them. When applying this theory to the school context, we consider that the demands that are difficult to be perceived by students to pass a specific subject may affect their level of stress and engagement (the same reasoning is also plausible for teacher demands). A moderate difficulty of demands, not too high, not too low, is recommended. According to Vygotsky’s theory, the degree of difficulty of a specific task (or demand) should be in the “zone of proximal development (ZPD),” which means that with the assistance of a more capable person, a child is able to learn skills or certain aspects of a skill that go beyond the child’s actual developmental level. The demands that students have to meet (studying demands) in order to pass a specific curricular subject (e.g., problem solving, assignments, oral presentations, lab work, study planning, etc.) are detailed in the subject’s planning and are subordinated to fulfill learning objectives. Students can obtain information about their required demands at the beginning of the course, mainly when the teacher introduces and explains the subject’s planning. The information from the evaluation system, such as tasks to do, level required to pass the subject, evaluation criteria, etc., is especially relevant for students.

Similarly, demands under teacher responsibility and their perceived difficulty may also influence teacher stress or engagement depending on their level of difficulty because, as we state before, demands can become stressing if they require a great deal of effort for the subject to meet them. The demands to be met by the teacher derive basically from the State’s Education Project, the District School Council and the school’s policy.

#### The Role of Support Resources in the Teaching–Learning Process

According to *The Job Demands-Resources Model (JD-R)* (Demerouti et al., 2001; Bakker and Demerouti, 2007), job resources refer “to those physical, psychological, social, or organizational aspects of the job that may reduce job demands and the associated physiological and psychological cost, are functional in achieving work goals, and stimulate personal growth, learning and development” (Hakanen et al., 2006, p. 497). When applying this theory to the school context, we assume that the perception of the supports/resources that the teacher and students are expected to be provided with to meet the required demands will influence their level of motivation and engagement. Thus supports/resources for teachers make their teaching work easier and help them to be more efficient to fulfill the stipulated instructional objectives (teaching demands). Conversely, the barriers/obstacles perceived by teachers not only prevent/hinder them from fulfilling the required objectives (teaching demands), but also cause them stress and burnout (Blase, 1982; Doménech and Gómez-Artiga, 2010).

#### External Support

Following the classification proposed by Schwarzer and Greenglass (1999), support can be internal or external. Internal support refers to students’ (e.g., self-efficacy) and the teacher’s (e.g., professional development) personal resources, and it will be treated in the following subsection. External support comes from the workplace, basically from administrative, social and didactic resources. Administrative support for teachers comes from school administrators (e.g., District School Council, supervisor, principal, etc.). Social support may come from either inside or outside the school. Referring to inside the school, supports for students come from teachers, peers, etc., whereas supports for teachers come from colleagues, psychologists, etc. Referring to outside the school, supports for students basically come from family and friends, whereas supports for teachers come from family and partners. Didactic resources refer to learning tools for students that are provided by teachers (e.g., computers, books, videos, etc.), as well as teaching tools for teachers (e.g., digital blackboard, computer, software, etc.) and school facilities (e.g., labs, library, gym, offices, etc.).

##### Support for students provided by the teacher

Regarding the supports for students provided by teachers, research has found that teachers shape students’ experiences in the classroom through both their teaching and interaction with students (Dietrich et al., 2015). Previous studies provided strong evidence linking teacher support and students’ academic emotions (see the meta-analysis conducted by Lei et al., 2018). The learning environment quality depends, to a great extent, on the simultaneous presence of the challenges offered and on the supports provided (Shernoff et al., 2016). Most authors have usually distinguished between affective/emotional or instructional/instrumental supports, but there is lack of consistency in the terminology used.

*(a) Emotional support.* According to previous studies (Patrick et al., 2011), definitions of *emotional support* usually include “students’ perceptions of trust, warmth, respect and love, as well as communication of empathy and care from their teachers” (Federici and Skaalvik, 2014, p. 21). Theoretically, some authors have distinguished between general and specific emotional support (Federici and Skaalvik, 2014). General emotional support refers to students’ general perception of the teacher as being warm, friendly, encouraging, etc., whereas specific emotional supports refer to emotional support in specific situations. Scales usually measure students’ general perception of their teachers as warm and friendly (Wentzel et al., 2010). A number of studies have centered on teachers being emotionally supportive, and have found emotional support to be related to high levels of intrinsic motivation (Katz et al., 2010), high levels of interest (Wentzel et al., 2010), and high levels of academic effort (Patrick et al., 2007; Reyes et al., 2012).

*(b) Instructional support.* According to previous studies (Suldo et al., 2009), definitions of *instructional support* usually include “students’ perceptions of being provided with instrumental resources and practical help” (Federici and Skaalvik, 2014, p. 22). This instructional help includes teacher behaviors, e.g., clarifying, correcting, modeling, questioning, etc., which contribute to understanding, problem solving or students developing skills (Malecki and Demaray, 2003). A number of studies have centered on teachers as being instructional supportive, and have found instructional support to be related to social, behavioral and academic outcomes (Malecki and Demaray, 2003). However, fewer studies have centered on instructional support than on emotional support. An effective teacher knows how to adapt the type of supports to the different kinds of tasks to be done and the demands to be met.

##### Support for students provided by peers

Peers can also provide help and support in developing social and academic competences (Wentzel et al., 2010); e.g., students interpret for each other teacher’s instruction and sometimes provide mutual assistance voluntarily. Therefore, peers support should also be taken into account.

##### Support for students provided beyond classroom context

Support for students can be provided beyond the classroom context, so the support that comes from outside the classroom (e.g., family, school, etc.) should also be considered. Jelas et al. (2016) examined adolescents’ perceptions of learning supports from parents, teachers and peers simultaneously, and their effect on student engagement. The data analysis was conducted by structural equation modeling. The results revealed that perceptions of learning supports explain and predict students’ affective, behavioral and cognitive engagement in school which, in turn, influences academic achievement. Empirical studies have focused on families and have proven the significant role of parents as contributors to school engagement and students’ performance at school (Bempechat and Shernoff, 2012). Family members who provide academic (e.g., assisting with homework) or motivational (e.g., recognizing effort and enhancing progress) supports help improve students’ academic performance (Jelas et al., 2016). Moreover, parents’ support and involvement may influence the way that their children perceive their own abilities or the way they value the subjects (Rodríguez et al., 2017).

#### Internal Support

As seen above, external support comes basically from the classroom and family context. Instead, internal support refers to students’ and the teacher’s personal resources.

##### Personal identity constructs as internal support/resources

Eccles (2009) underlines the importance of personal identity constructs (self-beliefs) which inform both students’ expectations of success and whether a task or subject is worth pursuing. In the same vein, Bandura (1993) argued that people’s personal variables, such as self-efficacy, influence the types of anticipatory scenarios they construct. Thus “students who have a high sense of efficacy visualize success scenarios that provide positive guides and supportive for performance” (p. 118). According to Eccles (2009), personal identity “can be conceptualized in terms of two sets of self-perceptions: (a) related to skills, characteristics and competences; (b) related to personal values and goals” (p. 78); e.g.: Who am I?; What are my weak and strong points?; What is important to me?; What do I value, etc. These are examples of questions related to personal identity. In contrast to social or collective identity, Eccles (2009) defines personal identity as “those aspects of one’s identity that serve the psychological function of making one feel unique” (p. 78). Positive self-beliefs (e.g., regarding to self-esteem, self-efficacy, self-concept, self-control, self-confidence, etc.) of the agents who intervene in the educational process (the teacher and students) act as internal support resources that will shape the initial perception that the teacher and students form of the educational situation context in terms of the resources/supports it offers to fulfill the required learning objectives. It is important to take into account the accuracy of perceptions; i.e., the degree of fit between the self-perceived (subjective) and real (objective) personal variables in order to design preventive or intervention programs to improve these variables. In short, it would be important to examine students’ self-perceptions (internal support resources) related to their personal variables from the beginning of the course. The self-beliefs formed by students and teachers related to their personal identity are grouped into the category called “internal personal variables”.

##### Previous research

Previous research (Doménech, 2006; Doménech et al., 2014, 2017; Abellán, 2016) conducted in the MOCSE field has already found some students’ personal variables that act as supports/resources to facilitate learning (e.g., academic self-efficacy and prior knowledge), which should be taken into account to build scales to assess internal supports. The research we are currently conducting with MOCSE will determine which personal variables have a greater predictive capacity on students’ intention to learn. All this information will help us to select which personal variables are to be considered in the future.

Before ending this section, we wish to underline some important ideas. First, by applying the Theory of Cognitive Appraisal (Lazarus and Folkman, 1984) to the educational setting it is plausible to assume that every time students (and also the teacher) face a new education situation, they make two evaluations (consciously or not) to determine if they have the necessary support/resources (internal and external) to face the requested demands. Second, the perception which students have formed of the educational setting, in terms of demands and support, at the beginning of the course is very important because it may condition their way of learning and engagement from the beginning. Third, students’ initial perception can be caused by (a) previous scholar experiences, (b) the information students already have (e.g., about the teacher, peers, etc.) (c) the information that arose on the first days of class when the teacher introduces the subject and explains the study program (evaluation requirements, type of demands, supports/resources provided, etc.), or when students check the teacher’s teaching style and personal characteristics. Fourth, although students’ initial perception tends to dynamically change during the T–L process (the longer the process, the more changes and fluctuations are expected to be produced), it is important to know how positive or negative students’ perception is at the beginning of the course given the implications it has for teaching and learning (details are provided below). Fifth, regarding support for students provided by the teacher, it is important to clarify that instructional supports primarily aim to achieve students’ subject domain (academic level of the classroom), whereas the teacher’s socio-emotional supports primarily aim to satisfy students’ psychological needs, such as autonomy, self-competence and feeling progress, relatedness and classroom integration, recognition, etc. (interpersonal and intrapersonal level of the classroom). How and when to provide these supports should be considered in teacher training programs. Finally, we wish to indicate that all these ideas focus on students, but can also apply to the teacher.

#### Relating Supports/Resources and Demands to Intention to Learn

**FIGURE 2:**
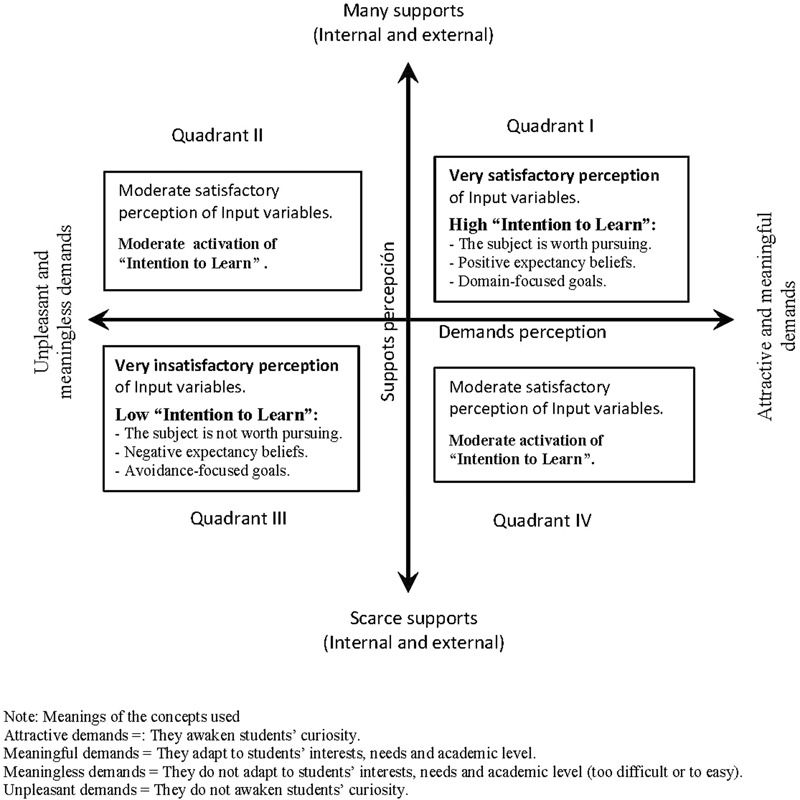
Ways of students perceiving the input variables and hypothesized predictions to intention to learn according to two bipolar orthogonal axes: One axis ranging between high supports-low barriers and low supports-high barriers, and the other axis ranging between attractive-meaningful demands and unpleasant-meaningless demands.

Generally, the *Job Demands-Resources Model (JD-R)* (Demerouti et al., 2001; Bakker and Demerouti, 2007) was used to explain employees’ work conditions in terms of demands and resources, and how these conditions relate to positive (e.g., engagement) and negative (e.g., stress and burnout) outcomes. Applying and adjusting the JD-R model to the school context implies identifying what the specific demands and support/resources are for teachers and students. Centered on students, supports/resources for students facilitate their learning and help them to fulfill the stipulated learning objectives (the major demand) and the other subordinate demands (studying demands) required to fulfill learning objectives. The main student supports come from the classroom and family context. According to Shernoff et al. (2016), the learning environment quality depends, to a great extent, on the simultaneous presence of the environmental challenge (reflected basically through educational demands), and on the provided supports (Shernoff et al., 2016). Students’ beliefs about themselves and their environment influence their motivation (Lin-Siegler et al., 2016). In order to examine students’ beliefs about themselves and their environment in terms of demands and supports/resources, we classified the perceptions that students make of demands and support variables into two orthogonal bipolar axes. One axis ranges between high supports-low barriers (the positive pole) and low support-high barriers (the negative pole). The other axis ranges between attractive-meaningful demands (positive pole) and unpleasant-meaningless demands (negative pole). The latter axis indicates to what degree demands connect with students’ interests, needs and academic level (meaningful *vs.* meaningless), and to what extent demands activate students’ curiosity (attractive *vs*. unpleasant). Based on the above rationale, we hypothesize that the perception of demands and support variables at the beginning of an instructional segment (e.g., task, theme, course, etc.), in terms of demands and support/resources, will predict students’ intention to learn. The conceptualization of intention to learn will be explained in the next sections. The formulated predictions are represented more specifically in **Figure 2**. As can be seen, quadrant I predicts the maximum activation of intention to learn; in contrast, quadrant III predicts the minimum activation of intention to learn. High difficult demands predict an expected stressful process during the course. Finally, we wish to point out that the perception of demands and support variables can also be influenced by prior academic experiences viewed by students in either the same educational setting or similar precursor courses (e.g., same subject, same teacher, etc.). A background of success will contribute to the perception of positive demands and support variables; in contrast, a background of failures will contribute to a negative perception. How the supports/resources and demands for teachers are related to intention to teach is currently under study.

### Component 2: Intention Activation Stage

Intention to learn is a complex construct in which multiple factors are involved. For us, intention to learn has the same meaning as motivation to learn. Intention is considered the immediate antecedent of action. We start from a basic premise: learning requires student’s intention to learn and the teacher’s intention to teach to be activated at the beginning of the educational process, and it has to remain active until the process ends. It is generated or activated on the first days of the T–L process in the agents (students and teacher) who take part in the educational setting, basically according to the information received from the environment on the demands required and the supports/resources offered when the course starts. However, it is assumed that intention to learn (and teach) does not remain constant, is ever-changing and fluctuates during the T–L process as a result of the constant (re)appraisals made by students of the internal and external influences they are exposed to in terms of the supports/resources and barriers/obstacles perceived to fulfill learning objectives (demands). Previous studies have indicated that this construct conditions all later educational processes from the beginning, and consequently learning outcomes (Doménech et al., 2014; Abellán, 2016).

Based on the pre-actional phase of the “Process Model of Student Motivation” (Dörnyei, 2000), and on a literature revision about achievement motivation in education, we propose two dimensions to operationalize and assess this construct: Expectancy-value beliefs (DI) and Goal setting (DII), which are associated with two mental and sequential processes. During the first process, initial wishes, desires and hopes are evaluated in terms of their chances of being fulfilled. During the second process, wishes, desires and hopes are transformed into goals. The latter represents the process through which learners specify and make a decision. The indicators selected for both dimensions derive from the three dominant theories in the contemporary literature of achievement motivation, all of which are grounded in a socio-cognitive perspective of motivation: the expectancy-value theory (Wigfield and Eccles, 2000; Eccles and Wigfield, 2002), Attribution theory (Weiner, 1992) and the achievement goal theory (Dweck and Legget, 1988; Nicholls, 1989; Ames, 1992; Wigfield and Eccles, 2000).

### Dimension I (DI): Expectancy-Value Dimension (EVD)

#### Indicator 1.1: Subject Value Beliefs

This construct is based on the assumption that the degree to which students’ believe that a subject or a task is worth pursuing is a key component for understanding students’ behaviors and learning outcomes (Liem et al., 2008). Although this concept seems relatively simple, it is not so because it has many conditioners. An object can have an intrinsic, extrinsic and instrumental value (as a step to fulfill a longer term goal). Eccles et al. (1983), Eccles and Wigfield (2002), and Eccles (2009) distinguished four components of task-value: attainment value, intrinsic value, utility value, and cost. They defined attainment value as the personal importance of doing the task well. Intrinsic value is the enjoyment that the individual gets from performing the activity (related to process expectancy). Utility value is determined by “how well a task relates to current and future goals, such as career goals” (Eccles and Wigfield, 2002, p. 120). Finally, these authors identified cost as a critical component of value, which was conceptualized as a negative determinant in engaging in a task due to, e.g., performance anxiety, fear of both failure and success, and the amount of effort needed to succeed (Eccles et al., 1983; Eccles and Wigfield, 2002). Prior studies (Cole et al., 2008) have found that students who perceive high task values make a greater effort and achieve more than those students who perceive low task values. In the same vein, Miller and Brickman (2004) argued that students only make their best effort and spend a substantial amount of time on mastering an academic task if they perceive it to be important and useful for them in the future.

#### Indicator 1.2: Expectancy Beliefs: Anticipatory Cognitive Motivators (ACM)

This concept is related to students’ (and the teacher’s) wishes, hopes and desires. If from the beginning of the course students (and the teacher) predict that their wishes, hopes and desires are going to be met in the current educational situation, they will transform them into goals (learning goals). Bandura (1993) argued that most human motivation is cognitively generated. “People motivate themselves and guide their actions anticipatory by the exercise of forethought” (p. 128). Bandura (1987) also indicated that the two basic capacities that largely explain human behavior are their *prediction* and *self-regulation* capacity. Individuals predict the consequences of future actions through prediction capacity. This capacity allows people to feel motivated and to regulate their actions in advance (self-regulation). Predictions can be associated with *underlying motivational processes* (Bandura, 1987). Motivational processes are activated by the learner (and the teacher) with a specific subject at the beginning of the course. **Table 2** shows the anticipatory cognitive motivators that we have selected for the MOCSE model and are suggested to be evaluated at the beginning of the course, after some days/weeks of class.

**Table 2:** Motivational constructs derived from the expectancy-value and the attribution theory.

Subject value and ACM^∗^	Constructs	Theory	Scales: Item example
(a) What does this subject mean for me?	**Subject value** Components: Attainment value, intrinsic value, utility value, and cost.	- Expectancy-Value Theory (Wigfield and Eccles, 2000) - Expectancy-Value model of achievement motivation (Eccles and Wigfield, 2002)	- “How useful is this subject for you?” - “How interesting is this subject for you?”
(b) Will I succeed in this subject?	**Success expectancy** Components: Self-efficacy expectancy and outcome expectancy.	- Expectancy-Value Theory (Wigfield and Eccles, 2000) - Expectancy-Value model of achievement motivation (Eccles and Wigfield, 2002)	- “Do you think you will be able to obtain good marks for this subject?” - “I will have the chance to show my capacities during the learning followed in this subject.”
(c) How will I feel in this subject?	**Process Expectancy** Students’ affective reactions with subject, teacher and peers.	- Expectancy-Value Theory (Wigfield and Eccles, 2000) - Expectancy-Value model of achievement motivation (Eccles and Wigfield, 2002)	- “Do you think you will feel well during the course?” - “Do you think you will feel well working this subject?”
(d) Is it worth studying and making the working effort for the benefits I will obtain in this subject?	**Cost expectancy** Profitability of work investment (effort and time).	- Subject value’s cost component. - Cost-Benefits Theory applied to education.	- “Will the time and effort you must invest to pass this subject be too much according to the importance you attach to this subject?” -“Will the time and effort you must invest to learn this subject be too much according to the interest it has for you?”
(e) To what extent does it depends on me, or not, to pass the subject?	**Controllability expectancy** Internal vs. external locus of control.	Attribution Theory (Weiner, 1992; Rotter, 1966)	- To what extent does it depend on me to pass or fail this subject? - To what extent does it depend on me to obtain good marks for this subject?

*^∗^ACM, anticipatory cognitive motivators.*

##### ACM from the expectancy–value theory

Expectancies for success, efficacy expectancies and outcomes expectancies were the more widely used in this tradition. Eccles and Wigfield (2002) defined expectancies for success as “individuals’ beliefs about how well they will do on upcoming tasks” (p. 119). It refers to students’ actual beliefs in their future expectancy for success; accordingly, expectancy for success is more future-oriented than simple self-perceptions of competence (Eccles and Wigfield, 2002). According to Eccles and Wigfield (2002), these expectancy beliefs are measured in a similar way to how Bandura’s (1997) personal efficacy expectations are measured. Bandura (1987) differentiated between “self-efficacy or efficacy expectations” and “outcome expectancy.” The former are defined as an individual’s belief in his/her own capability to accomplish a given task; the latter are defined as one’s belief that the effort one invests will lead to a desired outcome. The literature usually encompasses both self-efficacy constructs under the label “expectation for success” (Liem et al., 2008). Given the importance of students’ emotional state while they are doing a task or learning a subject, we decided to include an additional expectancy related to students’ affective reactions named *process expectancy.* We distinguished three components process expectancy beliefs: expected teacher–student, peer–student, and subject-student interaction. They refer to the feelings or affective reactions that students expect to experience in the new course, derived from the teacher–student, peers-student and subject–student relationships. Expectancy-value theorists consider the affective component to be crucial for understanding students’ engagement (Pintrich, 1989; Pintrich and De Groot, 1990). Indeed, nobody starts a task if they do not expect be feel well during the performance process (Pintrich and De Groot, 1990).

##### ACM from the attribution theory

The attribution theory explains students’ causal perception of their academic success and failure, and the emotional and motivational consequences. Attribution theorists emphasize the idea that individuals’ interpretations or causal attributions of their achievement outcomes (successes and failures), rather than motivational dispositions or actual outcomes, determine subsequent achievement strivings. First, theorists centered on the locus of control concept (Rotter, 1966) by distinguishing between internal and external locus of control. According to Rotter’s theory, one person should expect to succeed if (s)he feels in control of his/her own successes and failures (internal locus of control). Later, Weiner (1992) extended this idea and argued that the individual’s major causal attributions for achievement outcomes are ability, effort, task difficulty and luck. Weiner’s (1992) theory classified these attributions into three causal bipolar dimensions: locus of control, stability and controllability. The locus of control dimension refers to whether causes are located internally (internal locus of control) or externally (external locus of control) of the individual. The stability dimension refers to whether causes change over time or not (stability *vs.* instability). Controllability distinguishes the causes that one can control (e.g., skill or efficacy) from the causes that one cannot control (e.g., intelligence, mood or luck). In short, Weiner (1992) argued that the individual’s causal explanations for achievement outcomes are key motivational beliefs and they determine subsequent achievement strivings. Given the importance of causal attributional beliefs for engagement and motivation, these variables (especially the causal dimension of locus of control) were taken into account; they were not only oriented to students’ past experiences (mentioned in previous sections) where attributional beliefs are formed from reasoning to the past (retrospective reasoning), but also when attributional beliefs are formed from reasoning to the future, and play the role of anticipatory motivators.

### Dimension II (DII): Goal Setting Dimension (GSD)

This dimension refers to when students (and the teacher) make the decision, based on the previsions made from the anticipatory cognitive motivators, about how they will face the current situational setting.

#### Indicator 2.1: Achievement Goal Setting

Centered on students, this action is headed by the following question: What is my objective/purpose in this subject?, the answers lead to the “Achievement Goal Theory” (Dweck and Legget, 1988; Nicholls, 1989; Ames, 1992; Wigfield and Eccles, 2000). This theory posits that “the purposes that students hold for engaging in a specific academic task (i.e., their achievement goals) are an important antecedent to their achievement-related processes and outcomes” (Liem et al., 2008. p. 487). Thus, the Achievement Goal Theory (Dweck and Legget, 1988; Nicholls, 1989; Ames, 1992; Wigfield and Eccles, 2000) is proposed to explain and operationalize this dimension. Three types of achievement goal that have been usually studied are mastery, performance-approach and performance-avoidance (Skaalvik, 1997; Midgley et al., 2000). Those students who adopt a mastery goal focus on improving their competence and progress in an academic task/subject. The students who adopt a performance goal are concerned about the demonstration of competence shown by others. The students who adopt performance-avoidance goal wish to avoid social judgments and humiliation. The above classification can be completed with two more types of goals introduced by Alonso Tapia (2002) named self-worth and social recognition goals. Students who adopt the former want to get proud of their own performance. Students who adopt the latter type wish to obtain social recognition from others, such as teacher, parents, etc. Based on the two classifications exposed above, we have distinguished three broader groups of achievement goals according to two parameters: type of motivation (intrinsic *vs.* extrinsic) and type or reinforcement (positive vs. negative). Type I goals, based on intrinsic motivation. In this case the most important point for students is to improve their skills and progress (e.g., mastery goals or self-worth goals). Type II goals, based on extrinsic motivation and positive reinforcement. In this case the most important concern for students is to demonstrate their competence in obtaining social recognition or another reward (e.g., performance goals). Type III goals, based on extrinsic motivation and negative reinforcement. In this case the most important point for students is to avoid humiliation and embarrassment, and to protect their self-esteem (e.g., performance-avoidance goals). According to King and Mclnerney (2014) mastery and performance goals differ in terms of how competence is defined. “Students who pursue mastery goals define competence through interpersonal standards, while those who pursue performance goals define competence through normative comparisons with others” (p. 43).

#### Indicator 2.2: Avoidance Goal Setting

Finally, there is also the possibility that some students do not pursue any of the aforementioned goals. This occurs when students are not motivated to learn and make the minimum effort, or even try to avoid learning, which reflects passivity or inaction. Work avoidance represents the absence of an achievement goal (Elliot, 1999). For a more extensive description, see the study conducted by King and Mclnerney (2014), who examined the structure, antecedents and consequences of the avoidance goal construct.

To summarize, two main ideas are pointed out. First, the maximum activation of intention to learn is achieved when students believe that the subject is worth pursuing, make positive forecasts and adopt domain-focused goals. The main objective of these students is to master the subject, to learn and to progress. They are characterized by taking on difficult challenges, by striving to learn, and by getting actively involved (active coping) in the T–L process. Second, we use two dimensions to evaluate intention to learn (or motivation to learn), (a) the first dimension indicates the subject worth pursuing and chances of fulfillment, (b) the second dimension indicates the achievement goal-setting; i.e., the goals adopted by students. Students may be motivated to master the subject, obtain reinforcement, get good grades, demonstrate their worth, etc. Our proposal of the relationship expected between anticipatory cognitive motivators and goals is displayed in **Table 3**.

**Table 3:** Expected relationship between anticipatory cognitive motivators and goals.

	Type I goals (IM)	Type II goals (EM, +R)	Type III goals (EM, -R)	Avoidance goal (Amotivation)
ACM: Positive expectancy beliefs and high subject/contents value beliefs	+XX	+X	-X	-XX
ACM: Negative expectancy beliefs and low subject/contents value beliefs	-XX	-X	+X	+XX

*Goal description. Meanings of the symbols used: IM, Intrinsic motivation; EM, Extrinsic motivation; +R, Positive reinforcement; -R, Negative reinforcement. Relationship between goals and anticipatory cognitive motivators (ACM). Meaning of the symbols used: +XX, High positive relationship; -XX, High negative relationship; +X, Low positive relationship; -X, Low negative relationship.*

If we simultaneously consider both intention to teach and intention to learn, as they really take place in the classroom, any of the following basic possibilities could happen: (a) teacher’s teaching intention is optimal (high activation); (b) a teacher’s teaching intention is poor (low activation); (c) students’ learning intention is optimal (high activation); (d) students’ learning intention is deficient (low activation). To undertake a T–L quality process, it is necessary, but not enough, for intention to learn and intention to teach being optimal (see **Table 4**). The quality concept is explained in detail in the next section.

**Table 4:** Resulting interactions between intention to learn and intention to teach, and the expected predictions in T–L process quality.

Intentionality Student × Teacher		Intention to learn	
		Maximum activation	Minimum activation
Intention to teach	Maximum activation	Predict a **high quality** T–L–T process: Active, Efficient and Healthy (explained in next section).	Predict an intermediate quality of the T–L process.
	Minimum activation	Predict an intermediate quality of the T–L process.	Predict a **low quality** T–L process: Passive, Inefficient and Unhealthy.

*One necessary, but not sufficient, condition for a quality T–L process is that the teacher’s intention to teach and students’ intention to learn are high.*

Finally, we wish to put forward some important ideas before ending this section.

(a) The MOCSE model emphasizes the importance of taking into account this construct (intention to learn and to teach) at the beginning of any T–L process some days/weeks after the course starts. The data obtained so far (Doménech, 2006, 2011a; Doménech et al., 2014; Abellán, 2016) seem to confirm that students’ initial motivation predicts and explains students’ involvement in mastering the subject.
(b) Motivation evolves gradually through a complex psychological process that involves basically initial goal setting, intention activation and planning (*pre-actional phase*) before action implementation. We are aware that the components we propose to evaluate intention to learn are a necessary, but not sufficient, condition to completely explain action.
(c) We wish to underline that intention to learn (and intention to teach) does not remain constant during the T–L process. On the contrary, it is subjected a dynamically changing and fluctuating pattern as a result of constant (re)appraisals of the internal and external influences (in terms of the supports/resources and barriers/obstacles perceived to fulfill learning objectives) that students are exposed to.
(d) Finally in long-term processes, motivation to do something basically involves intention activation, making decisions and action implementation in this sequential order, although these steps follow on from each other almost simultaneously. This means that it is practically impossible to separate pre-actional and actional phases in complex processes (Dörnyei, 2000).

In short, the first two components of the model (appraisal phase and intention activation), plus the educational action plan, represent the pre-actional decisional phase, and are responsible for learner and teacher decisions about how to deal with a new educational process. Students decide in this phase if they first want to engage in order to master the school subject or not. So we call the variables from components 1 and 2 “predictive variables” because they are able to predict students’ engagement from the beginning of the course (Doménech, 2006, 2011a; Doménech et al., 2014; Abellán, 2016).

Basically, there are three time points when the agents involved in the learning-teaching process make important decisions about the course, which are also known as macrodecisions (microdecisions are made about the theme or task). The most important time point is the period at the beginning of the course, but the time points that correspond to the second and third trimesters are also important (in Spain, school courses last three terms, and each term lasts 3 months). The decisions made at these two time points are mainly triggered by knowledge about a new element: the results that students obtain when they end each trimester, which are provided on a report card.

## Actional Phase: Teaching– Learning Process

The actional phase (represented by component 3) covers those variables relating to the teaching and learning strategies undertaken by the teacher and students to achieve learning objectives. Specifically, it refers to how the three key elements (teacher, content, and learner) interact during the T–L process conducted in the classroom with a specific subject matter. Following Vigotsky, we can state that the teacher and students interact through content (instrumental mediator), while students interact with content mainly through the teacher (social mediator). The interaction of the three key elements (teacher–content–students) is crucial for quality learning. Based on this assumption, in this section we present an integrative approach of the T–L process, operationalized and defined by specific quality indicators.

### Quality of Learning

There are a number of ways to understand the quality of a product or service: Quality as Exceptional, Quality as Perfection or Consistency, Quality as Fitness for Purpose, Quality as Value for Money, Quality as Transformation, etc. For more details, see Harvey and Green (1993). In my opinion, the better perspectives to be applied to education are “Quality as Perfection or Consistency” and “Quality as Transformation.” The first quality approach centers on the process and establishes the specifications that must be followed to achieve perfection. The motto that defines this perspective is “getting thinks right the first time.” The second quality approach centers on participants’ qualitative changes and transformation, which means enhancing the participant and provoking as many changes as possible during the T–L process. Applying both notions to education implies following during the T–L process (both the teacher and students) the principles that psycho-educational research has demonstrated as being effective to generate as many changes as possible in students in the cognitive and socio-affective domains by taking the fixed learning objectives as a reference to conduct instruction.

Taking the interaction of the three key elements (teacher, content, and students) as starting point, a high quality T–L process first requires the active and simultaneous participation of the three key elements throughout the T–L process. Nevertheless, each has a different degree of responsibility in the different phases that make up the process (Rivas, 1997, 2003). So, cognitive and physical activity and the interaction among participants in a specific curricular subject are an essential condition of the quality T–L process. The second requirement of is to provoke the most marked transformation possible among students in their cognitive and socio-effective domains. The quantity and quality of the academic results acquired (in terms of changes and transformation) are related to T–L process efficiency. Finally, the third requirement refers to the teacher having to enjoy teaching and students having to enjoy learning. The latter can only be possible if the teacher and students experience positive emotions while implementing the T–L process. Positive emotions in the classroom are related to the psychological health of both teachers and students. These three characteristics (active, efficient and healthy) influence each other. Below we explain in more detail the three traits that best define a high quality T–L process.

#### Active

Active learning is usually defined by authors as the learning that requires students engaging cognitively and meaningfully with materials (Bonwell and Eison, 1991) to get involved with the information presented (analyzing, summarizing evaluating) rather than just passively receiving it (King, 1993). According to this conceptualization, active learning is understood as “cognitively engaged.” However this term (active learning) has also been considered as a multifaceted construct, from motivational, behavioral and emotional perspectives (Chi and Wylie, 2014; Fredricks et al., 2004). From a motivational perspective, active learning focuses on either the precursor attitude or the interest in getting involved in learning tasks. From a behavioral perspective, active learning focuses on student actions, such as how often students attend class or do their homework, etc. Finally from an emotional perspective, active learning focuses on affective reactions to teachers, peers, etc. In the current work, active learning is used as cognitive and behavioral engagement. Taking Lazarus and Folkman’s (1984) theory as a reference, it is plausible to consider that if students and the teacher perceive a stimulating and supportive environment, they will wish to get engaged (cognitively and behaviorally) in the T–L process, and will consequently develop active coping strategies that focus on the problem (e.g., seeking social support, confronting the problem, etc.) that aim to manage/overcome any problems and challenges that may arise. On the contrary, if they perceive a threatening environment they will wish, mainly, to protect themselves from negative emotions, and will therefore very likely develop passive strategies that focus on emotion (e.g., escape-avoidance, distancing, etc.). It would be desirable for both students and teachers to adopt active coping strategies (problem-focused coping), engaging and striving to overcome the problems or challenges that may arise throughout the educational process. Teachers can promote active learning by creating a more active learning environment (Bonwell and Eison, 1991; Fink, 2013) by, for example, designing significant lessons and activities that overcome passive learning. The ICAP framework (Chi and Wylie, 2014) provides guidelines for teachers as to how to promote active learning.

#### Efficient

A T–L process is considered efficient when it provokes the most marked transformation possible in students in their cognitive and socio-effective domains. This will be possible if the psycho-educational principles and specifications derived from the empirical research proposed by the literature are followed by teachers and students. The principles and specifications capable of facilitating learning were interpreted basically in terms of demands and supports/resources. Teacher support contributes to maintain student “executive motivation” which, in turn, affects learning outcomes. Demands for students are related to learning objectives, tasks and contents. Demands must be assumed by students who, besides teacher support, also need to apply effective strategies to accomplish the required demands (“getting things right the first time”), all of which subordinate to achieve learning objectives. Therefore, effective actions and strategies from the teacher and students are needed to meet learning objectives. The transformation achieved by students during the T–L process conducted in a specific subject matter can be operationalized by calculating the difference between the students’ starting point in the pre-instructional phase (students’ initial state) and the post-instructional phase (students’ final state).

#### Healthy

It is important that the teacher enjoys teaching and that students enjoy learning. This means having to focus on the process as much as on the results, or even more. When focusing on the educational situation as a stage where the T–L process takes place, it is important to study and identify the process elements that could affect (positively or negatively) the psychological health of teachers and students. Traditionally, the research carried out in this field has focused more on studying negative emotions (teaching discomfort, stress, burnout, etc.) rather than on positive ones. However, this approach has changed in recent years with the rise of positive psychology.

From the psychological point of view, we understand a healthy educational process as one that produces in agents or participants (the teacher and students) positive emotions; on the contrary, if the emotions experienced by the involved subjects are mostly negative, it is considered an unhealthy process (be aware that triggers can also originate from the external contexts directly connected to the educational situation, such as family and school).

Teaching is highly emotional work (Marchesi, 2007) given the constant interactions among the three key elements (teacher, content and students) throughout the T–L process. Based on MISE, we particularly stress the importance of the Personal Interactions dimension (dimension three) as most of the emotions experienced by the agents involved (the teacher and students) are activated in this phase. Based on the Control-Value Theory of Achievement Emotions (Pekrun et al., 2007), we must take into account the processes of “appraisals control” (efficacy beliefs, expectations, and responsibilities) and “value appraisals” (perceived task value and results) since, according to this theory, they are important antecedents of achievement emotions. A healthy educational process will provide well-being for the teacher and students which, in turn, will affect the quality of education. Finding the instructional keys that can contribute to teacher and student well-being is a challenge that we are currently working on. Measurement instruments assessing students’ achievement emotions are provided by Pekrun and his colleagues (see Pekrun et al., 2005, 2011).

### Quality Indicators of the Teaching–Learning Process

Finally, we present an integrative approach of the T–L process, where the indicators were organized and classified using an adapted and reduced version of the “Educational Situation Instructional Model” (MISE, acronym in Spanish) designed by Rivas (1997, 2003). The resultant version called MISE-4D is composed of four dimensions: Instructional design execution (DI), Personal interactions (DII), Knowledge acquisition (DIII), and Evaluation (DIV). Some indicators which integrate the four dimensions are fundamentally the teacher’s responsibility, while others are students’ responsibility. Finally, others have a shared responsibility. The resulting primary structure and configuration is displayed in **Table 5**.

**Table 5:** The basic structure of MISE-4D (adapted from Rivas, 1997, 2003), and the teacher/student’s responsibility in each indicator.

MISE-4D, Dimensions and indicators	Responsability
**(D) (I) Instructional design execution: Supports for student**	
I. 1.1. Instructional support.	Teacher
I. 1.2. Emotional support.	Teacher
I. 1.3. Didactic support resources.	Teacher
**(D) (II) Personal interactions: Classroom climate**	
I. 2.1. Teacher-student interaction: Classroom management.	Teacher-Student
I. 2.2. Peer interaction: Partnership and support.	Student
I. 2.3. Affective reactions experienced.	Teacher-Student
**(D) (III) Knowledge acquisition: Learning processes**	
I. 3.1. Evolutionary parameters: conditioning and activators.	Student
I. 3.2. Previous knowledge: contents and conceptions.	Student
I. 3.3. Motivational and attentional processes.	Student
I. 3.4. Learning strategies and approaches.	Student
I. 3.5. Dedication: time and effort.	Student
**(D) (IV) Evaluation: Feedback to the T–L process**	
I. 4.1. Evaluation during the T–L process: Formative.	Teacher
I. 4.2. Evaluation after the T–L process: Summative or final.	Teacher
I. 4.3. Psychological individual effect: Anxiety/Stress.	Student

Currently, we investigate the contribution of the MISE-4D indicators to the three main features (active, efficient and healthy) that define the quality of the T–L process. In a recent study (Doménech, 2017, Unpublished) carried out with a sample of 127 educational psychology students we explored the MISE-4D factorial validity in the university context and the relationship between the MISE-4D variables (factors extracted from the exploratory factor analysis) and some criteria variables about students’ involvement, emotions and achievement. A preliminary version of the MISE-4D questionnaire was obtained with good Cronbach’s alpha values for the factors extracted. The correlational analyses provided interesting clues to identify which MISE-4D variables are involved in the quality of the T–L process in the university context. **Table 6** shows the results obtained in the study in detail. In order to find regularities in similar educative situations (subjects) and at the same educational level, further research is needed to identify the role played by the MISE-4D variables in each specific context. Specifically, investigating the contribution of the MISE-4D variables on the three features (active, efficient and healthy) that define the quality of a T–L process at different levels/subjects is suggested

**Table 6:** MISE-4D Factorial validity and bivariate correlations between the factors extracted and some criterial variables about university students’ involvement, emotions and achievement (*N* = 127).

Dimensions	Indicators	Factors extracted	α	Progress	Anxiety	Course satisfaction	Participation	Class attendance
DI: Instructional design execution Items = 23	I. 1.1. Instructional support.	- F1. Support student’s autonomy (4 items)	0.82	0.20^∗^	-0.21^∗^	0.38^∗∗^	ns	0.24^∗^
(73.25% of variance)		- F2. Support to understand teacher explanations (4 it.)	0.82	0.33^∗∗^	Ns	0.57^∗∗^	Ns	0.30^∗∗^
		- F3. Support subject utility (3 items)	0.89	0.29^∗∗^	ns	0.37^∗∗^	ns	ns
	I. 1.2. Emotional support.	- F4. Support student motivation (3 items)	0.87	0.20^∗∗^	ns	0.41^∗∗^	ns	0.29^∗∗^
		- F5. Deactivating negative emotions (4 items)	0.89	0.33^∗∗^	-0.24^∗^	0.39^∗∗^	0.21^∗^	0.38^∗∗^
	I. 1.3. Didactic support resources.	- F6. Didactic resources provided (5 items)	0.80	ns	-0.29^∗∗^	0.29^∗∗^	ns	25^∗^
DII: Personal	I. 2.1. Teacher-student interaction	- F1 Classroom management (5 items)	0.81	ns	ns	0.38^∗∗^	ns	0.21^∗^
interactions Items = 17	I. 2.2. Peer interaction	- F2 Peer interaction (4 items)	0.82	ns	ns	ns	ns	ns
(69.73% of variance)	I. 2.3. Affective reactions experienced.	- F3 Enjoy learning (4 items)	0.93	0.43^∗∗^	ns	0.77^∗∗^	0.21^∗^	0.46^∗∗^
		- F4. Proud of progress (4 items)	0.85	0.53^∗∗^	ns	0.56^∗∗^	0.51^∗^	0.59^∗∗^
DIII: Knowledge	I. 3.1. Evolutionary parameters	(Not considered)						
acquisition Items = 22	I. 3.2. Previous knowledge	- F1. Prior knowledge (3 items)	0.88	0.24^∗^	-0.36^∗∗^	0.32^∗∗^	ns	ns
(73.10% of variance)	I. 3.3. Motivational/attentional processes.	- F2. Intrinsic-executive motivation (3 items)	0.82	0.46^∗∗^	ns	0.64^∗∗^	0.23^∗^	43^∗∗^
	I. 3.4. Learning strategies and approaches.	- F3. Help-seeking (4 items)	0.76	0.32	ns	0.47^∗∗^	0.37^∗^	64^∗∗^
		- F4. Avoid memorizing (4 items)	0.80	0.42	ns	0.44^∗∗^	0.33^∗^	39^∗∗^
		- F5. Problem-focused approach (4 items)	0.91	0.27^∗∗^	ns	0.21^∗^	0.21^∗^	ns
	I. 3.5. Dedication: time and effort.	- F6. Time and effort (4 items)	0.81	0.61^∗∗^	ns	0.48^∗∗^	0.32^∗∗^	0.44^∗∗^
DIV: Evaluation	I. 4.1. Formative evaluation.	- F1. Formative evaluation (5 items)	0.89	0.29^∗∗^	-0.20^∗^	0.51^∗∗^	ns	0.22^∗^
Items = 16	I. 4.2. Summative or final evaluation.	- F2. Final evaluation (6 items)	0.88	0.42^∗∗^	ns	0.50^∗∗^	ns	ns
(69.35% of variance)	I. 4.3. Psychological individual effect: Anxiety/Stress.	- F3. Anxiety/stress caused by the exam (5 items)	0.89	ns	0.49^∗∗^	ns	ns	ns

*^∗^p < 0.05, ^∗∗^*p* < 0.01.; ns, not significant. Criterial variables (reported by students): Scale used: Ranged from 0 (nothing) to 10 (very high). (1) Students’ achieved progress (1 item). “Indicate the progress you have made in this subject to date” (2) Students’ anxiety experienced during the course (1 item). “Indicate the level of anxiety/stress you have experienced during the course to date” (3) Course satisfaction (1 item). “Indicate your level of overall satisfaction with the theoretical classes-problems to date” (4) Student participation in class. (1 item). “Indicate your level of active participation in the theoretical classes (asking, volunteering, contributing ideas, etc.) during the course to date.” (5) Class attendance (1 item): “Indicate your level of attendance to educational psychology classes to date”.*

## Reflectional Phase: Product

### Results and Satisfaction

The product phase refers to learning outcomes. Student achievement and satisfaction are two of the most important learning outcomes of students, and are also considered key indicators of education quality.

Regarding academic achievement, the quality of students’ results may be conditioned by teachers’ beliefs about learning, which are reflected at the beginning of the course in the learning objectives formulation (students’ demands). We understand learning as a change in students to move from an initial state to a final one (Rivas, 1997, 2003). Traditionally teachers have placed more emphasis on the cause of quantitative and information-conceptual changes rather than on more useful and effective formative changes to solve problems and to make decisions. However in recent years, a paradigm shift in this direction has been promoted by experts and educational leaders. In this sense, the type of results to be achieved at schools/education centers has been redefined in terms of generic and specific skills/competences, which relate more with the challenges posed by today’s society. Thus the formative change is identified by the kind of learning we wish to achieve in the students formulated in terms of skills/competences, and is operationalized through learning outcomes. The final goal of all T–L processes is to accomplish the desired results specified in terms of learning objectives.

Satisfaction is considered both an outcome of the T–L process, and an important requirement for successful learning (Sinclaire, 2014). It would be desirable for a sense of satisfaction to be perceived by both the teacher and learners. Students should experience satisfaction with the results they obtain and with the T–L process followed. Student satisfaction is conceptualized by Sweeney and Ingram (2001) as “the perception of enjoyment and accomplishment in the learning environment” (p. 2), and by Lo (2010), as “students subjective perceptions of how well a learning environment supports academic success” (p. 48). The role played by the teacher in terms of the (instructional and emotional) supports provided may contribute to increase student satisfaction (enjoyment and accomplishment). Previous research has identified a number of factors that contribute to student satisfaction, among which we wish to highlight interaction (Wu et al., 2010) and teacher communication (Parayitam et al., 2007), considered by students to be two important teacher supports.

As seen in **Figure 1**, the T–L process undertaken during a course with a specific curricular subject provides feedback to the appraisal and intention activation stages, which favor/facilitate the self-regulation of the model to improve outcomes and satisfaction.

### Improving Results and Satisfaction

#### Actions Centered on the Classroom Level

Three main actions, under teachers’ responsibility, should be undertaken during the course to improve learning outcomes and student-teacher satisfaction.

##### First, intention to learn evaluation (pre-actional phase)

In accordance with the exposed rationale in previous sections (understanding quality education from a preventive perspective), we wish to underline the importance of taking into account the variables through which intention to learn is operationalized during the first days/weeks of the education process. Therefore, the first action to perform aims to evaluate *intention to learn variables* at the beginning of the T–L process because this will provide teachers with valuable information about the extent to which students will be engaged in studying and working on a specific subject. Thus, if necessary, preventive or corrective instructional measures may be applied in time when motivation deficiencies are detected to increase students’ motivation during the educative process. If the evaluated intention to learn is deficient, it would then be recommendable to identify the demands and support variables responsible for this shortcoming. Besides correcting deficiencies, diagnosis evaluation also allows the teacher to adjust/fit support to students’ characteristics at the beginning of the course. As we state before, in order to increase intention to learn, attractive and meaningful demands should be designed, and emotional and instructional supports (from teachers, peers, parents, etc.) should be provided.

There are basically three time points in which the agents (students and their teacher) involved in the T–L process make decisions about the course, which we call macrodecisions (microdecisions are made about the theme or task). The most important time point is the period when the course begins, but the time points that correspond to the start of the second and third trimesters are also key. The decisions made at both these time points are triggered mainly by knowledge of a new element: the results that students obtain at the end of each trimester, provided on a report card. So if we want to know the evolution of students’ macrodecisions throughout the course, it will be necessary to make evaluations of their intention to learn at the three aforementioned time points. A practical guide to improve intention to learn/motivation to learn and the scales for secondary students can be found in Doménech and Abellán (2017).

##### Second, evaluation of the T–L process (actional phase)

**FIGURE 3:**
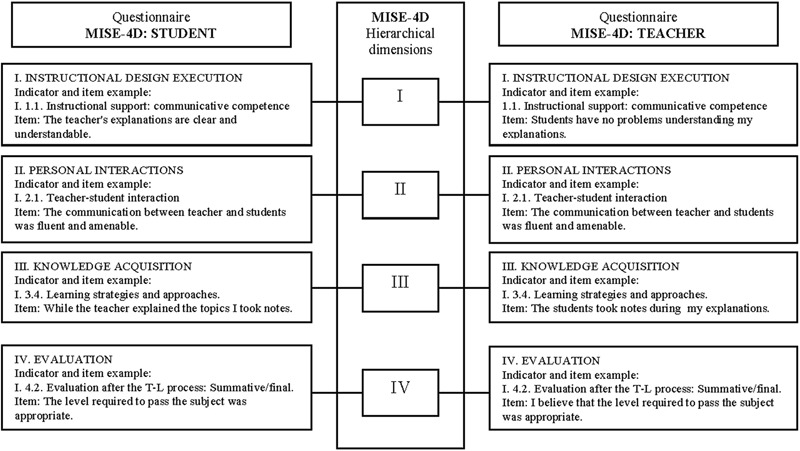
Item examples of the MESE-4D teacher and MSE-4D student’s questionnaires.

The second action centers on the evaluation of the T–L process (formative evaluation) that the teacher should conduct to improve the quality of the process undertaken, and carrying this out about halfway through the course is recommended, The aim of this evaluation is to detect educational process’ weak and strong points, to correct weak aspects in time and to persevere with strong features. MOCSE provides two MISE-4D instruments designed to be used by the teacher and students to carry out the T–L process evaluation. So based on both the MISE-4D dimensions and their corresponding indicators (see **Table 5**), we constructed two questionnaires (MISE-4D for the teacher and MISE-4D for students) to evaluate the T–L process from the teacher and student perspectives (see Doménech, 2011a, 2012). This procedure allows us to know to what extent the teacher and students have assumed their part of responsibility during the T/L process, and to compare the information reported by the teacher and students about the same referents; i.e., the MISE 4D dimensions and indicators (see **Figure 3**). Furthermore, it should be pointed out that the MISE-4D is a versatile model as it allows indicators to be adapted to a specific educational setting, while the core model structure is preserved (dimensions and indicators); i.e., any teacher can adapt the MISE-4D questionnaires to the subject that he/she teaches without changing the core model structure. So MISE becomes a valuable tool for teacher formative evaluation as it provides them with empirical data from teacher and students perspective throughout the T–L process.

##### Third, product evaluation (reflectional phase)

Finally, the learning outcomes and satisfaction experienced about the outcomes and the process followed should be considered and evaluated. The aim of this evaluation is to first know to what extent the learning objective has been fulfilled and, second, to know the student satisfaction reported of both the results they have obtained and the T–L process followed. This evaluation provides the teacher with feedback about the model’s functioning. It will allow the teacher to reflect retrospectively by introducing changes to improve the model’s components for subsequent courses.

#### Actions Centered on the School Level

The same procedure and actions can be implemented at the school level. Previously, teachers should be trained in how to use the MOCSE model in the classroom. The empirical data obtained through MOCSE procedures can provide the scientific basis to design effective programs for schools adapted to different levels of education and subjects. It is also a useful tool that can be employed by policy makers and educational leaders to design prevention and intervention programs to increase learning outcomes and satisfaction at schools.

## Conclusion

MOCSE is an instructional model that provides a conceptual framework capable of explaining the functioning of an educational setting in an integrative way. To design the model, contributions made from several relevant psycho-educational theories have been taken into account. This work has focused mainly on the learner and on an educational situation/subject matter (macroscopic level), but what is presented herein can also be applied to an instructional unit or a task (microscopic level).

This proposal introduces a new perspective into the existing literature that will allow researchers to make progress in studying educational setting functioning. It also provides a methodology and the necessary instrumentation to be used as a tool to guide research and reflection in the classroom. Reflective practice is the basis to acquire high teaching skills (Osterman and Kottkamp, 1993/2004; Zeichner and Liston, 1996; Cole and Knowles, 2000; Larrivee, 2000; Jay, 2003).

The research done to date within the MOCSE framework has centered basically on students, specifically on examining the relation that links intention to learn, the learning process and academic results. The obtained findings have provided some interesting clues to explain why students decide to make an effort and get involved in their learning while others have decided the contrary, and show no interest at all to learn. Moreover, the model’s integrated view allowed us to design valid instruments to identify the weak and strong points of the T–L process undertaken in the classroom from the students and teacher perspectives. This approach promotes the teacher’s reflection and subsequent actions to be taken in order to improve the quality of the process followed with a specific subject.

However, more investigation is needed. First, to identify what are the best supports to meet the required demands for teacher and students in different educational levels and curricular context. These findings may provide a scientific basis to design prevention and intervention programs that aim to improve students’ engagement and learning outcomes. Second, since this work has centered mainly on learners, some important questions on the teacher still remain unsolved and need to be investigated in the future to complete the MOCSE conceptual framework, such as: What are the commonest demands to be met by teachers and what supports can be received from inside and outside the classroom?; What are the roles played by demands and the supports for teachers in activating their intention to teach? What are the suitable intentions to teach indicators to define and operationalize this construct?; What are the specific actions of the teacher’s responsibility and of learners’ responsibility that contribute to the quality of the T–L process?, etc. Finally, investigating the role of both teachers and students simultaneously within the MOCSE framework is a future challenge.

## Author Contributions

FD-B: contributed to the conception and the design of the work, review the research and final writing.

## Conflict of Interest Statement

The author declares that the research was conducted in the absence of any commercial or financial relationships that could be construed as a potential conflict of interest.
